# Enrichment of Alkaloids from *Cinnamomum camphora* Seed Kernels Using Macroporous Resin: Adsorption/Desorption Behavior, Process Optimization and Scale-Up Study

**DOI:** 10.3390/foods15061054

**Published:** 2026-03-17

**Authors:** Rongping Fu, Xianghui Yan, Zheling Zeng, Yujing Yang, Pinpin Zhang, Yuling Lin, Deming Gong, Ping Yu

**Affiliations:** 1 School of Chemistry and Chemical Engineering, Nanchang University, Nanchang 330031, China; frp_2121@163.com (R.F.); 17698319443@163.com (Y.Y.); zhang_20010206@163.com (P.Z.); 2 State Key Laboratory of Food Science and Resources, Nanchang University, Nanchang 330047, China; xianghui_y@163.com (X.Y.); dgong01@gmail.com (D.G.); 3 College of Food Science and Technology, Nanchang University, Nanchang 330031, China; 4 School of Pharmacy, Nanchang University, Nanchang 330031, China; 15679592193@163.com; 5 New Zealand Institute of Natural Medicine Research, 8 Ha Crescent, Auckland 2104, New Zealand

**Keywords:** macroporous resin, magnoflorine, lindoldhamine, N,N-methyldomesticinium, scale-up, antioxidant

## Abstract

The *Cinnamomum camphora* seed kernel (CCSK) shows great promise as a natural source of bioactive alkaloids. However, there is little data about recovering alkaloids from CCSK by-products after oil extraction using an aqueous method. This study aimed to establish an efficient technology for enriching CCSK alkaloids (including magnoflorine, lindoldhamine and N,N-methyldomesticinium) using macroporous resin technology. The results showed that XR918C resin was the most suitable adsorbent due to its high adsorption/desorption capacity for CCSK alkaloids. The adsorption process was best described by Langmuir isotherm models and pseudo-second-order kinetics; it was spontaneous and physical in nature. The optimum procedure for CCSK alkaloids enrichment using XR918C resin was as follows: for adsorption, the injection flow rate and sample volume were 2.0 BV/h and 7.0 BV, respectively; for desorption, the eluent type, elution flow rate and volume were 80% ethanol, 2.0 BV/h and 6.0 BV, respectively. Furthermore, the scale-up of the CCSK alkaloid enrichment process was performed under optimal conditions. Following the 10-fold scale-up enrichment, the content of CCSK alkaloids was raised 4.41-fold, with a recovery rate of 89.19 ± 0.01%. After nine regeneration cycles, the efficiency of the XR918C resin remained stable, indicating its good reusability. In addition, CCSK alkaloids exhibited strong in vitro antioxidant activity. This study provides a useful reference for the industrial-scale enrichment of CCSK alkaloids.

## 1. Introduction

It has been established that a plethora of secondary metabolites are synthesized by plants, which facilitate their growth and reproduction [1]. These secondary metabolites encompass a wide range of chemical compounds, including alkaloids, phenols, steroids, glycosides, tannins, terpenes and antitoxins [2]. Numerous studies have indicated that these secondary metabolites exhibit diverse biological activities, rendering them valuable in terms of their potential applications in the domains of human and animal health [3]. In particular, alkaloids are an important class of secondary metabolites [4], which are defined as organic compounds characterized by a cyclic structure incorporating one or more basic nitrogen atoms within their molecular framework [5]. Furthermore, alkaloids demonstrate a range of pharmacological properties, encompassing anti-inflammatory, antioxidant, antiviral and antineoplastic activities. Valipour et al. [6] found that isoquinoline alkaloids exhibited anti-inflammatory and antiviral effects, and these alkaloids can be used as powerful natural anti-SARS-CoV-2 drugs. Mombeini et al. [7] reported that berberine had the antioxidant and prevention effect of cyclophosphamide nephropathy. These properties are mechanistically associated with their interactions with specific biomolecular targets [8].

The *Cinnamomum camphora* (L.) Presl., commonly known as the camphor tree, is a species of evergreen broad-leaved plant that is distributed extensively throughout Southern China, particularly in the southern area of the Yangtze River [9]. It plays an important role in both the native flora and the art of landscaping in China [10]. Notably, the annual yield of *C. camphora* seeds is over 11 million tons in China. However, *C. camphora* seeds have caused serious contamination in both urban and rural environment due to a lack of development [11]. Our previous study showed that *C*. *camphora* seed kernels (CCSKs) contained 48–63% medium-chain oil and 18–19% protein [12]. In addition, CCSK was found to be rich in bioactive substances, including alkaloids, phenols and steroids [13,14]. In particular, our previous research showed that magnoflorine, lindoldhamine and N,N-methyldomesticinium were the primary alkaloids in CCSK [14]. These alkaloids exhibited various health benefits, including anti-diabetic, anti-inflammatory and antibacterial activities. For instance, Cherku et al. [15] found that magnoflorine exhibited anti-diabetic properties and prevented weight loss in diabetic mice, producing effects comparable to those of metformin treatment. Guo et al. [16] found that magnoflorine had an anti-inflammatory effect, reducing the expression of TNF-α, IL-6 and other pro-inflammatory cytokines. Osmakov et al. [17] reported that lindoldhamine exhibited anti-inflammatory properties in mice. Taken together, these data suggest the significant potential application of CCSK alkaloids in functional foods and biological medicines. Therefore, the development of CCSK alkaloids is extremely important.

The most common methods currently used for extracting alkaloids include liquid–liquid extraction [18], solid-phase extraction [19] and precipitation at a basic pH [20]. However, these methods have several disadvantages, including solvent consumption, high cost, and inapplicability to industrial processes. In contrast, macroporous resin (MAR) is well-suited to industrial production and has been employed to enrich alkaloids from plant-based ingredients, such as *Coptidis rhizoma*, *Euodiae fructus* [21] and peony seed meal [22]. The high selectivity and adsorption capacity of MAR make it potentially valuable for use in the pharmaceutical and food industries. Additionally, the unique chemical composition of CCSK means that the residual aqueous solution after CCSK oil extraction using the aqueous extraction (AE) method contains loads of nutrients, including proteins, polyphenols, alkaloids and polysaccharides [23]. However, the refinement of alkaloids from the CCSK aqueous solution using MAR technology has yet to be reported.

Therefore, this study aimed to enrich the alkaloids in the CCSK aqueous solution using MAR chromatography. Firstly, the adsorption and desorption behaviors of 16 commercial MARs with regard to CCSK alkaloids were compared. The optimum enrichment conditions were then determined. Eventually, the enrichment process was scaled up in the laboratory. This research supplies a foundation for the industrial enrichment of CCSK alkaloids.

## 2. Materials and Methods

### 2.1. Materials

Naturally mature *C. camphora* seeds were collected from Nanchang University (Nanchang, China) in January 2025. Ten MARs (X-5, HPD-700, HPD-200, HPD-300, HPD-722, HPD-BJQH, HPD-400, HPD-450, HPD-750 and NKA-9) were purchased from Baoen Adsorption Material Technology Co., Ltd. (Cangzhou, China), two MARs (XR918C and XR930C) were purchased from Shanghai Xuner Chemical Industry Technology Co., Ltd. (Shanghai, China), two MARs (YKDH-2 and YKDH-9) were from Tianjin Yunkai Resin Technology Co., Ltd. (Tianjin, China), and two MARs (LS-300 and LS-300B) were from Xi’an Lan Shen New Material Technology Co., Ltd. (Xian, China), and their physical properties are shown in Table S1. Magnoflorine, lindoldhamine and N,N-methyldomesticinium standards (HPLC ≥ 98%) were prepared in our laboratory (China patent number: ZL202510329638.8[P]). The NMR spectra of the three marker alkaloids are shown in Supplementary Material (Figures S1–S3). Formic acid and methanol were obtained from Aladdin Biochemical Technology Co., Ltd. (Shanghai, China).

### 2.2. Pretreatment of MAR

The MAR was pretreated using the method reported by Wang et al. [24]. In short, the MAR was first soaked in anhydrous ethanol for 24 h, after which the resin was washed with distilled water until the alcohol concentration measured by an alcoholometer was zero. It was sequentially soaked in solutions of 5% NaOH (*w*/*v*) for 6 h and 5% HCl (*v*/*v*) for 6 h. Then it was purged with distilled water until neutral. Finally, the MAR was soaked in absolute ethanol for later use.

### 2.3. Preparation of CCSK Aqueous Solution by AE Method

The AE method was used to treat the CCSK, as described by Zhu et al. [11]. Briefly, to produce a slurry, the CCSK was combined with distilled water at a 1:4 (*w*/*w*) ratio, poured into a colloid mill for 5 min, and then centrifuged at 4800 rpm for 10 min to obtain the CCSK aqueous solution. The intermediate aqueous phase was collected and stored at 4 °C for later use.

### 2.4. Determination of Alkaloids Before and After MAR Enrichment

The alkaloids in the CCSK aqueous solution before and after MAR enrichment were quantitatively analyzed using an Agilent 1260 Infinity III LC system (Agilent Technologies, Inc., Santa Clara, CA, USA) equipped with a variable wavelength detector. Prior to analysis, the freeze-dried samples of CCSK aqueous solution before and after MAR enrichment were dissolved in HPLC-grade methanol at a concentration of 1 mg/mL, followed by filtration using a 0.22 μm organic membrane. The sample was then separated at 25 °C using a gradient elution procedure on an Amethyst C18-H reversed-phase column (4.6 × 250 mm, 5.0 μm; Sepax Technology Co., Ltd., Suzhou, China). The flow rate was 1.0 mL/min, and the mobile phase consisted of 0.1% (*v*/*v*) formic acid in water (A) and methanol (B). The elution procedure was as follows: 0–8 min, 5–20% B; 8–28 min, 20–50% B; 28–40 min, 50–85% B; 40–45 min, 85–5% B; and 45–55 min, 5% B. The alkaloids in the CCSK aqueous solution before and after MAR enrichment were detected at 280 nm and identified by comparing their retention times and UV spectra with those of the standard substances, magnoflorine, lindoldhamine and N,N-methyldomesticinium.

The external standard method was used to quantify the alkaloids. The equations of the calibration curves for magnoflorine, lindoldhamine and N,N-methyldomesticinium are shown below. They are as follows: Magnoflorine: Y = 6.5269x + 7.4868 (R^2^ = 0.9998, *n* = 6); Lindoldhamine: Y = 4.3462x − 1.2552 (R^2^ = 0.9996, *n* = 6); and N,N-Methyldomesticinium: Y = 4.1959x − 1.2136 (R^2^ = 0.9999, *n* = 6), where Y represents the area of the peak and x represents the concentration of the alkaloids. The content of alkaloids in the CCSK aqueous solution before and after MAR enrichment was calculated by summing the contents of magnoflorine, lindoldhamine and N,N-methyldomesticinium. The results were expressed on a dry weight basis to ensure consistency and comparability.

### 2.5. Resin Screening

In a 50 mL conical flask, 40 mL of the CCSK aqueous solution and MAR (1.00 g) were combined. Then, the flask was shaken in the thermostat vibrator (ZWY-21-2C; Zhicheng Analysis Instrument Manufacturing Co., Ltd., Shanghai, China) at 180 rpm and 25 °C for 3 h. Subsequently, the MAR was cleaned with distilled water. Under the same conditions, the MAR was then desorbed with 40 mL of 80% ethanol. The most suitable MAR was selected, based on its adsorption and desorption capacity for CCSK alkaloids. The HPLC method described in Section 2.4 was used to calculate its adsorption and desorption capacity. The relevant calculation formulas (Equations (S1)–(S4)) are shown in the Supplementary Materials. XR918C resin was chosen for the next experiment by analyzing the static tests results of 16 MARs.

### 2.6. Adsorption Kinetics

In a 100 mL conical flask, 60 mL of the CCSK aqueous solution was combined with the chosen XR918C resin. The mixture was then shaken at 180 rpm and 25 °C. The alkaloid concentration in the solution was measured using the HPLC method described in Section 2.4 at the following time intervals: 0, 0.5, 1.0, 2.0, 3.0, 4.0, 5.0, 6.0, 7.0, 8.0, 9.0 and 10.0 h. The relevant calculation formulas (Equations (S5)–(S7)) of the adsorption kinetic model are shown in the Supplementary Materials.

### 2.7. Adsorption Isotherms

CCSK aqueous solutions (30 mL) with various doses of alkaloids (0.309, 0.638, 0.821, 0.975, 1.31 and 1.65 mg/mL) were combined with the chosen XR918C resin (0.5 g). The conical flasks were then shaken for 3 h at 180 rpm and different temperature (25, 35 and 45 °C). Subsequently, the resin adsorption capacity of alkaloids was calculated. The relevant calculation formulas (Equations (S8)–(S10)) of the adsorption isotherms model are shown in the Supplementary Materials.

### 2.8. Calculation of Adsorption Thermodynamic Parameters

To analyze the adsorption process of CCSK alkaloids onto XR918C resin from a thermodynamic perspective, a comprehensive thermodynamic analysis was conducted, encompassing the calculation of three pivotal parameters (ΔG, ΔH and ΔS). The relevant calculation formulas (Equations (S11) and (S12)) of adsorption thermodynamic parameters are shown in the Supplementary Materials.

### 2.9. Dynamic Adsorption/Desorption Tests

To ascertain the most effective parameters for the purpose of enriching alkaloids from the CCSK aqueous solution, a series of dynamic adsorption and desorption tests were conducted. These tests were carried out using a chromatographic column with a bore diameter of 16 mm and an extent of 300 mm. The chromatographic pillar was filled with pretreated resin, which had a height-to-diameter ratio of 14 (1 BV was 45 mL). To ascertain the impact of the flow rate on the efficacy of adsorption, the CCSK aqueous solution (2.45 mg/mL) was subjected to the chromatography column at a rate of 1 to 4 BV/h.

To study the impact of the desorption rate on desorption efficiency, desorption experiments of sample-laden columns at three different flow rates (2.0, 3.0 and 4.0 BV/h) were performed with the best eluent. The dynamic curve of C_t_/C_0_ over time was plotted, and the dynamic adsorption capacity of XR918C resin for CCSK alkaloids at various flow rates was calculated. Additionally, Thomas, Adams–Board and Yoon–Nelson dynamic models were used to fit the dynamic breakthrough data [25]. The calculation formulas were as follows Equations (1)–(4):

(1) $$\ln\left(\frac{C_{t}}{C_{0}}\;-\;1\right)=\frac{K_{\mathrm{TH}}Q_{e}m}{Q}\;-\;K_{\mathrm{TH}}C_{0}t$$

(2) $$\ln\frac{C_{t}}{C_{0}}=K_{\mathrm{AB}}C_{0}t-N_{0}h\frac{K_{\mathrm{AB}}}{\nu }$$

(3) $$\ln\left(\frac{C_{t}}{C_{0}-C_{t}}\right)=K_{Y}t-K_{Y}\tau$$

(4) $$Q=n\frac{V}{60}\;(n=2,\;3,\;4)$$

where C_0_ (mg/mL) and C_t_ (mg/mL) represent the concentration of CCSK alkaloids at initial and time t; K_TH_ (mL/mg/min), K_AB_ (mL/mg/min) and K_Y_ (min^−1^) are the constants of reaction rates; Q_e_ represents the maximum adsorption amount of CCSK alkaloid; m (g) represents mass of adsorbent; Q (mL/min) and ν (cm/min) represent flow rate of CCSK alkaloid solutions; N_0_ (mg/mL) represents the adsorption amount of packed-sorbent per unit volume; h (cm) represents bed depth of sorbent; τ is the moment at which C_t_ of CCSK alkaloid was up to 50% of C_0_; and V (mL) represents the chromatographic pillar volume.

### 2.10. Scale-Up of Resin Column Chromatography

A 10-fold scale-up in the processing volume of the dynamic enrichment test was performed under optimum enrichment conditions (mentioned in Section 2.9). The test was carried out using a laboratory-scale column with 3.5 cm inner diameter and 60 cm length, which was filled with pretreated XR918C resin. The diameter-to-length ratio of the fixed bed and the experimental conditions were the same as in Section 2.9.

### 2.11. Reusability Evaluation of XR918C Resin

According to Wang et al. [26], the reusability of XR918C resin was evaluated under the optimal conditions through repeated adsorption and desorption experiments in CCSK alkaloids. Desorption was performed using 80% anhydrous ethanol, after which the resin was washed with distilled water until the alcohol concentration measured by an alcoholometer was zero. The adsorption and desorption experiments were then repeated nine times.

### 2.12. Determination of Antioxidant Activity of CCSK Alkaloids Before and After Enrichment

The antioxidant activity of CCSK alkaloids before and after enrichment was evaluated using DPPH and ABTS free radical scavenging assays [27].

#### 2.12.1. DPPH Radical Scavenging Activity

Briefly, the sample solutions at different CCSK alkaloid concentrations (10, 20, 40, 60, 80 and 100 μg/mL) were prepared by dissolving the freeze-dried powder in absolute ethanol. Then, 150 μL of DPPH solution (40 μg/mL, dissolved in absolute ethanol) was mixed with 50 μL of the sample solution. The mixture was then incubated in the dark at room temperature for 30 min, after which its absorbance (A_1_) was measured at a wavelength of 517 nm using a microplate reader (ReadMax 1900; Flash Spectrum Biological Technology Co., Ltd., Shanghai, China). The absorbance of a mixture (A_0_) of 150 μL of DPPH solution and 50 μL of distilled water and the absorbance of a mixture (A_2_) of 150 μL of absolute ethanol and 50 μL of sample solution were measured under the same conditions. The DPPH radical scavenging activity (%) of the sample was then calculated using the following formula:

(5) $$\mathrm{DPPH}\;\mathrm{radical}\;\mathrm{scavenging}\;\mathrm{activity}\;(\%)\;=\;\left(1-\frac{A_{1}-A_{2}}{A_{0}}\right)\;\times \;100\%$$

#### 2.12.2. ABTS Radical Scavenging Activity

A solution of 7 mM ABTS was mixed with a solution of 2.45 mM potassium persulfate (K_2_S_2_O_8_) in the dark at room temperature for 12–16 h to produce an ABTS free radical solution. Then, the obtained ABTS free radical solution was diluted with ethanol until its absorbance at 734 nm reached 0.70 ± 0.02 (TU-1950; Purkinje General Instrument Co., Beijing, China). Next, 150 μL of the ABTS radical solution was mixed with 50 μL of the sample solution at various concentrations (10, 20, 40, 60, 80 and 100 μg/mL; prepared as in Section 2.12.1). After incubating the mixture in the dark at room temperature for 30 min, the absorbance (A_1_) was measured at 734 nm. The absorbance of the mixed solution (A_2_) containing 150 μL of distilled water and 50 μL of the sample solution and of the mixed solution (A_0_) containing 150 μL of the ABTS free radical solution and 50 μL of distilled water were measured under the same conditions. The ABTS free radical scavenging activity of the sample was calculated according to the following formula:

(6) $$\mathrm{ABTS}\;\mathrm{radical}\;\mathrm{scavenging}\;\mathrm{activity}\;\left(\%\right)\;=\;\left(1\;-\;\frac{A_{1}\;-\;A_{2}}{A_{0}}\right)\;\times \;100\%$$

### 2.13. Statistical Analysis

All data were presented as the mean ± standard deviation (SD) from at least three replicate experiments. Differences between groups were assessed using one-way ANOVA and Tukey’s test (SPSS Statistics 22 software). *p* < 0.05 was considered statistically significant.

## 3. Results and Discussion

### 3.1. Resin Screening

**Figure 1:**
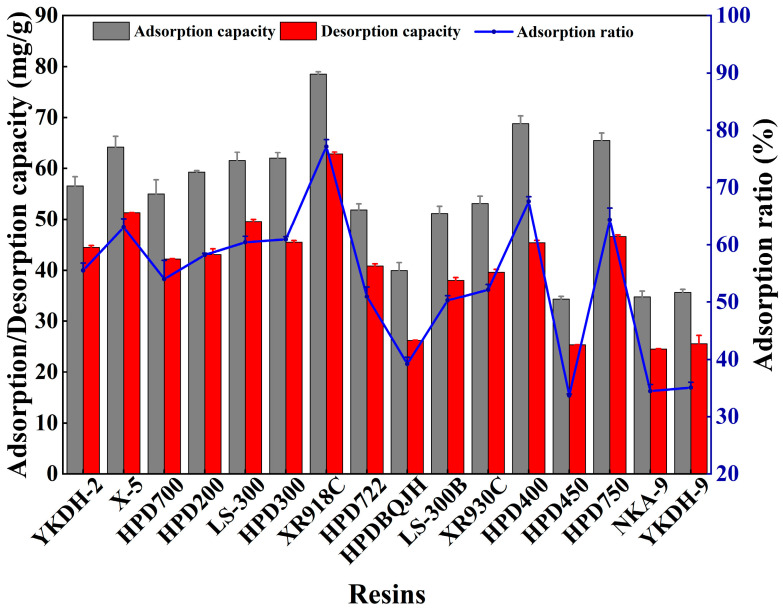
Effects of different MARs on the adsorption capacity (gray column), desorption capacity (red column) and adsorption ratio (blue line) of CCSK alkaloids.

The physicochemical characteristics of the adsorbents significantly impact their adsorption and desorption capacities. The ideal adsorbents should exhibit high selectivity for the adsorbates as well as high adsorption and desorption capacities [28]. MAR chromatography is a satisfactory technique due to its large surface area, high selectivity and high adsorption/desorption capacities [29]. There are a large variety of commercial MARs with various physicochemical characteristics. This study examined the adsorption ratios and adsorption/desorption capacities of sixteen MARs for the enrichment of CCSK alkaloids (including magnoflorine, lindoldhamine and N,N-methyldomesticinium). As shown in Figure 1, compared with the weak, medium and polar MARs, nonpolar MARs had greater adsorption capacity for CCSK alkaloids, indicating that the polarity of the MAR was a critically important factor influencing its adsorption capacity for CCSK alkaloids. Notably, among the eight non-polar MARs, XR918C exhibited the highest adsorption capacity (78.55 ± 0.46 mg/g), followed by X-5 (64.21 ± 2.14 mg/g), HPD-300 (62.03 ± 1.11 mg/g) and LS-300 (61.54 ± 1.64 mg/g). Among the four weak polar resins, XR930C exhibited the highest adsorption capacity (53.13 ± 1.45 mg/g). Among the three semi-polar MARs, HPD-750 exhibited the highest adsorption capacity (65.51 ± 1.46 mg/g). These differences may be due to the different physical properties of MARs. For example, the larger pore size of X-5 enables it to combine with CCSK alkaloids in the active sites of the resin’s pores more effectively, compensating for the disadvantage of its small surface area. The larger specific surface area of HPD-750 enables it to absorb more CCSK alkaloids. These results suggested that the adsorption capacity of MAR was not only affected by the polarity, but also by the specific surface area and pore size.

The MAR desorption capacity is also an important factor affecting the enrichment process [30]. The desorption capacities of the eight non-polar MARs were as below: XR918C (62.83 ± 0.38 mg/g) > X-5 (51.32 ± 0.01 mg/g) > HPD-750 (46.62 ± 0.3 mg/g) > HPD-722 (40.84 ± 0.46 mg/g) > YKDH-9 (25.58 ± 1.46 mg/g). Combining these results with those for adsorption capacity showed that the greater the adsorption capacity of the MAR, the greater its desorption capacity. In particular, XR918C resin demonstrated the optimal adsorption and desorption performance, which is possibly because XR918C resin has a large specific surface area and appropriate pore size. Therefore, XR918C resin was chosen for subsequent experiments.

### 3.2. Adsorption Kinetics

**Figure 2:**
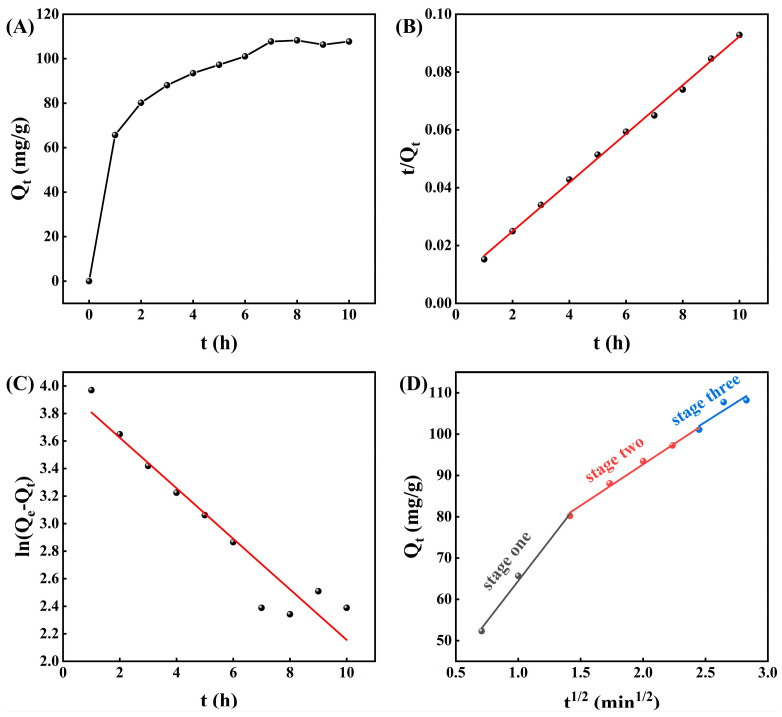
Adsorption kinetics curve (**A**) and linear correlations based on the pseudo-first-order (**C**), pseudo-second-order (**B**) and intraparticle diffusion (**D**) models for CCSK alkaloids on XR918C resin. Q_e_ (mg/g) represents the total amount of CCSK alkaloids adsorbed at equilibrium; Q_t_ represents the total amount of CCSK alkaloids adsorbed onto the resin at time t.

The relationship between the adsorption rate and the adsorption equilibrium time of the adsorbents can be described by adsorption kinetics [31]. This is important for designing experiments and scaling them up for practical applications. As shown in Figure 2A, the XR918C resin showed a rapid increase in its adsorption capacity for alkaloids from CCSK during the initial hour (*p* < 0.05), followed by a slower increase within 1–7 h, with no change within 7–10 h. The quick raise in adsorption capacity may be due to the substantial number of adsorption sites on the surface of XR918C resin in the initial stage, leading to the adsorption of a large quantity of CCSK alkaloids within the first hour.

The pseudo-first-order model (PFO), the pseudo-second-order model (PSO) and the intra-particle diffusion model were used to describe the adsorption process and elucidate the adsorption mechanism. Figure 2B,C showed the plots of PFO and PSO. The predicted adsorption capacity of the PSO (118.6240 mg/g) was more closely aligned with the experimental value (108.2260 mg/g) and significantly higher than that of the predicted value of the PFO (54.1414 mg/g) (Table 1). Additionally, the R^2^ of the PSO (0.9976) was higher than that of the PFO (0.9086). Therefore, it can be deduced that the PSO may be more suitable for the CCSK alkaloid adsorption process than the PFO.

The adsorption of adsorbates onto resins is generally divided into three stages: external film diffusion (stage I), intra-particle diffusion (stage II), and adsorption of adsorbates at active sites (stage III) [32]. The adsorption of CCSK alkaloids by XR918C resin occurred in three stages (Figure 2D). The straight line from Q_t_ to t^1/2^ did not pass through the origin, indicating that adsorption involved both external membrane diffusion and intra-particle diffusion [33]. The results suggests that resistance of resin itself exerts a significant influence on the adsorption of CCSK alkaloids.

### 3.3. Adsorption Isotherms

**Figure 3:**
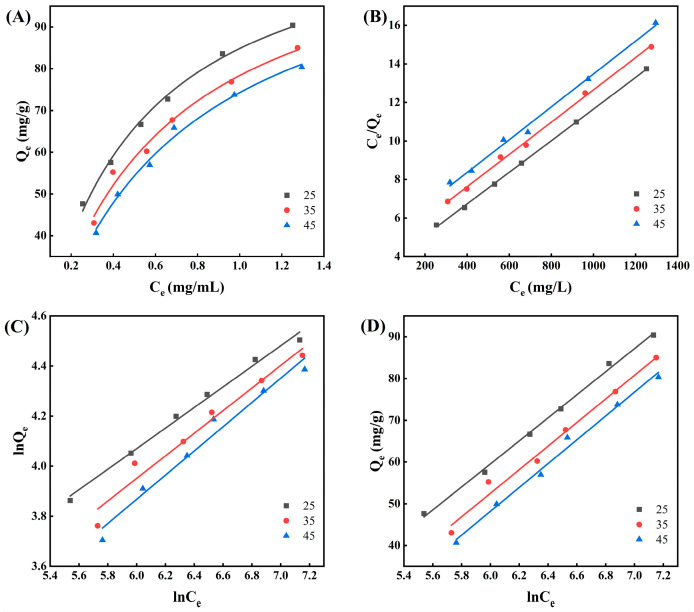
Adsorption isotherms (**A**) and linear correlations based on the Langmuir (**B**), Freundlich (**C**) and Temkin (**D**) models for CCSK alkaloids on XR918C resin at 25, 35 and 45 °C.

Adsorption isotherms are defined as the relationship between the equilibrium adsorption capacity of an adsorbent and the equilibrium concentration at a constant temperature [34]. Studying adsorption isotherms is important for understanding the interactions, optimizing performance parameters and conserving energy. As shown in Figure 3A, an increase in the adsorption capacity of XR918C resin for CCSK alkaloids was proportional to an increase in CCSK alkaloid concentration. Additionally, an increase in temperature was found to decrease the equilibrium adsorption capacity of resin, indicating that higher temperatures are not conducive to adsorption processes.

The adsorption of CCSK alkaloids onto the XR918C resin at 25, 35 and 45 °C were evaluated using the Langmuir, Freundlich and Temkin models (Figure 3B–D and Table 2). The Langmuir model is a well-known isothermal model used to evaluate the performance of adsorbents. The model describes the adsorbent surface as having uniformly distributed adsorption sites and clarifiers that, when a monolayer is adsorbed onto a uniform surface, there are no interactions between adjacent molecules [35]. As shown in Table 2, the value of predicted adsorption capacity (Q_m_) gradually decreased as the temperature rose, indicating that elevated temperatures were not conducive to the adsorption process. Additionally, as the temperature increased, the K_L_ value gradually decreased, suggesting that CCSK alkaloids could be attached to the resin surface at a lower temperature. The K_L_ value was between 0 and 1, indicating favorable adsorption isotherms for XR918C resin [36]. The Freundlich model is an empirical equation used to calculate the non-ideal or multi-layer adsorption of an adsorbate onto a heterogeneous surface [37]. The K_F_ value decreased as the temperature increased (Table 2), indicating that the adsorption was exothermic. Moreover, the value of 1/*n* ranged between 0 and 1, suggesting that the adsorption of CCSK alkaloids onto XR918C resin was straightforward [38]. The Temkin model demonstrates that the binding energy decreases approximately linearly with an increase in the binding number of adsorbents. As shown in Table 2, the A_T_ value gradually decreased with increasing temperature, indicating that an increase in temperature resulted in a decrease in binding force. Overall, the Langmuir model was deemed the most appropriate model for describing the adsorption of CCSK alkaloids on XR918C resin.

### 3.4. Adsorption Thermodynamics

**Figure 4:**
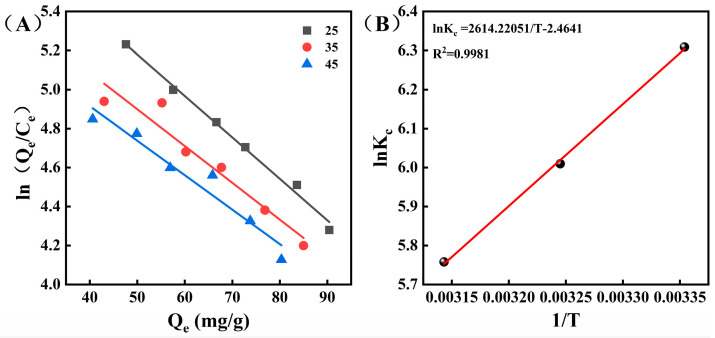
Plots of ln(Q_e_/C_e_) versus Qe at 25, 35 and 45 °C (**A**); plots of lnKc versus 1/T (**B**).

Adsorption thermodynamics can reveal detailed information about changes in the structure and internal energy of the adsorbent after adsorption, as well as providing insights into the adsorption process [39]. Figure 4A showed the plot of ln(Q_e_/C_e_) versus Q_e_, and K_eq_ was determined by extrapolating Q_e_ to zero. Figure 4B showed the plot of lnK_eq_ versus 1/T, and ΔH and ΔS were calculated from its intercept and slope, respectively [40]. As shown in Table 3, the value of ΔG was negative, indicating that the adsorption process was spontaneous and thermodynamically feasible. Additionally, the greater the absolute value of ΔG, the lower the temperature at which CCSK alkaloids could be adsorbed by XR918C resin [41]. A negative value of ΔH indicated that the adsorption of CCSK alkaloids onto XR918C resin was exothermic. Additionally, the absolute value of ΔH was much less than 40 kJ/mol, suggesting that the adsorption was physical [14,42]. Furthermore, the negative ΔS value indicated that the randomness of the solid–liquid interface decreased when CCSK alkaloids were adsorbed onto XR918C resin [43].

### 3.5. Dynamic Test

#### 3.5.1. Dynamic Breakthrough Curves

**Figure 5:**
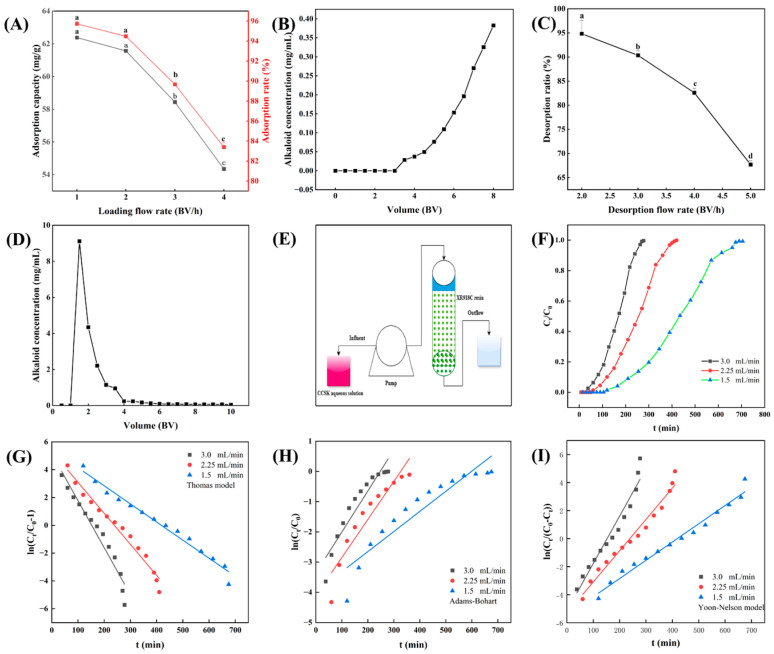
XR918C resin-based chromatographic enrichment of CCSK alkaloids. Loading flow rate (**A**), breakthrough curves (**B**), desorption flow rate (**C**) and desorption curves (**D**); dynamic adsorption column adsorption (**E**); plotting C_t_/C_0_ versus t (min) in column experiments (**F**); and fitting results of dynamic sorption process using Thomas model (**G**), Adams–Bohart model (**H**) and Yoon–Nelson model (**I**). The different letters (a–d) indicate significant differences (*p* < 0.05).

Dynamic breakthrough curves are plotted by monitoring changes in adsorbate concentration over time at the outlet of the adsorption column. This allows the adsorption process to be analyzed quantitatively and provides guidance on its application. To determine the optimal flow rate and volume of the CCSK aqueous solution, the adsorption breakthrough curves of XR918C resin for CCSK alkaloids at 1, 2, 3 and 4 BV/h were analyzed. The adsorption rate decreased as the adsorption flow rate increased (Figure 5A). However, there was no significant difference between the adsorption rates at 1 BV/h and 2 BV/h (*p* > 0.05). Consequently, to enhance the adsorption efficiency, the sampling flow rate was set at 2 BV/h. Additionally, the leakage point (1/10 of the initial concentration) was reached at a sample volume of 7 BV as the volume of the sample increased (Figure 5B). Therefore, under the optimal dynamic adsorption conditions, the loading flow rate was 2 BV/h and the loading volume was 7 BV.

#### 3.5.2. Dynamic Elution Curves

Studying elution curves helps determine the appropriate flow rate and volume for elution, which both directly affect the purity and yield of the product. To enrich alkaloids from the CCSK aqueous solution, the XR918C resin was initially subjected to a water wash, employing 2 BV of water to ensure the removal of any impurities that were not adsorbed. To determine the optimal flow rate and volume for desorption, the resin was then desorbed using 80% ethanol as the desorption agent at flow rates of 2, 3, 4 and 5 BV, respectively. As shown in Figure 5C, the desorption ratio gradually decreased as the flow rate increased. Therefore, a flow rate of 2 BV/h was used. As shown in Figure 5D, the concentration of the desorption substance initially increased and subsequently decreased as the volume of the desorption solution increased. When the volume of the desorption solution exceeded 6 BV, there were almost no CCSK alkaloids detected. To reduce the ethanol consumption, 6 BV was selected as the optimal desorption volume.

#### 3.5.3. Breakthrough Curve Modeling

Accurately predicting a breakthrough curves’ properties is essential for the successful design and fabrication of an adsorption column [44]. In order to accurately evaluate the feasibility of using XR918C resin for practical applications, the Thomas, Yoon–Nelson and Adams–Board models were explored to describe and predict the dynamic adsorption behavior (Figure 5E). As shown in Figure 5F, both the effluent breakthrough time and the CCSK alkaloid concentration depended heavily on the flow rate; specifically, the lower the flow rate, the longer the breakthrough time.

The Thomas model is useful for predicting the relationship between effluent concentration and time, which has been widely used in adsorption column studies. The Thomas model was used to fit the experimental data in order to determine the adsorption capacity (q_e_) and the rate constant (K_TH_), which were calculated according to the intercept and the slope of the fitted equation. As shown in Figure 5G and Table 4, an increase in the flow rate from 2.0 to 4.0 BV/h resulted in an increase in K_TH_ from 0.0059 to 0.0154 mL/mg/min. Conversely, the trend in the q_e_ value changed (from 50.3952 to 36.2767 mg/g), consistent with a study on methyl blue adsorption [45]. The experimental penetration curve data showed a high correlation with the fitted Thomas model (0.94 < R^2^ < 0.97), indicating that the Thomas model accurately describes CCSK alkaloid adsorption on the XR918C resin adsorption column.

The values of N_0_ (maximum adsorption capacity) and K_AB_ (coefficient of mass transfer) were determined using the intercept and slope of the fitted Adams–Board equation (Figure 5H and Table 4). An increase in flow velocity results in an increase in the K_AB_ value, indicating that the system’s kinetics are primarily driven by mass transfer. Conversely, an increase in flow velocity results in a decrease in the N_0_ value. Of the three models, the Adams–Board model had the worst fit, with R^2^ values ranging from 0.86 to 0.89. This result indicated that the Adams–Board model was not suitable for describing CCSK alkaloids adsorption on the XR918C resin column.

The Yoon–Nelson model can be used to predict the relationship between the time taken for an adsorbate to become exhausted at a given concentration and the adsorption process [46]. The values of K_Y_ (rate constant) and τ (time required for 50% CCSK alkaloids breakthrough) were determined from the intercept and slope of the Yoon–Nelson model-fitting equation (Figure 5I and Table 4). As the flow rate increased, K_Y_ increased and τ decreased. A decrease in τ indicated that the consumption rate of the adsorption column would accelerate with an increase in flow velocity, which was undesirable for the adsorption process. At the same time, the smaller the τ value, the better the performance of adsorption column. As shown in Table 4, the Yoon–Nelson model fitted well (0.94 < R < 0.97). Therefore, the data from the adsorption column experiment conformed to the Yoon–Nelson model.

### 3.6. Qualitative and Quantitative Analysis of CCSK Alkaloids

**Figure 6:**
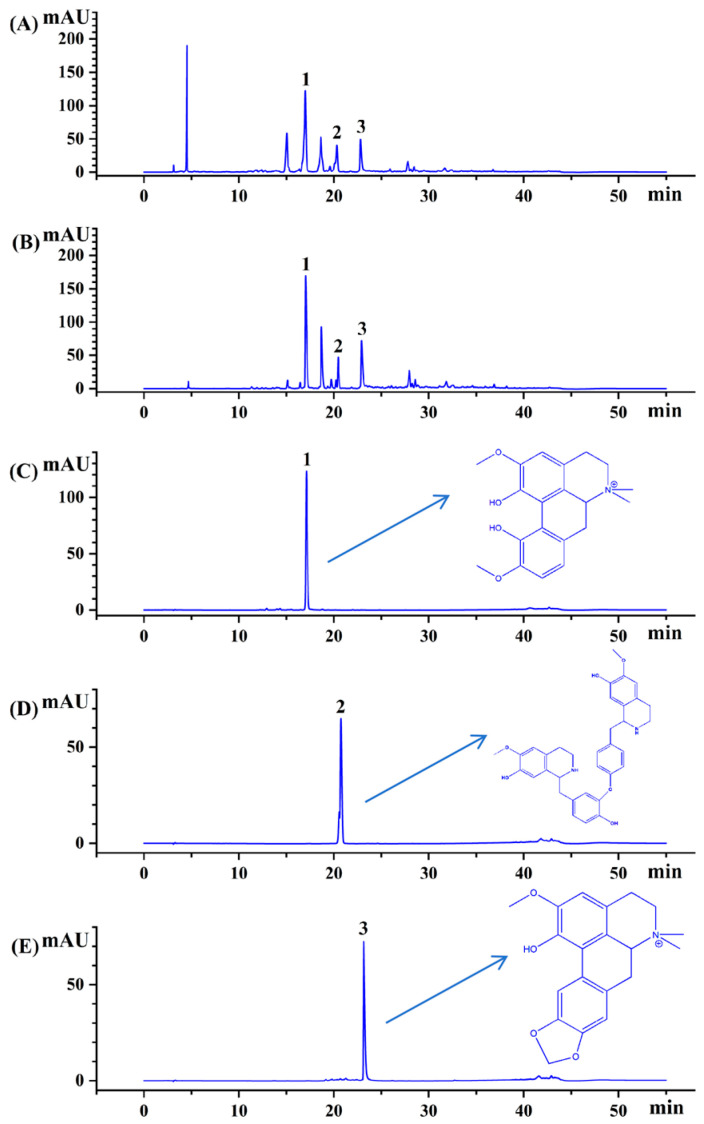
HPLC chromatograms of CCSK alkaloids before (**A**) and after (**B**) XR918C resin enrichment. Three alkaloids: magnoflorine (**C**), lindoldhamine (**D**) and N,N-methyldomesticinium (**E**).

As shown in Figure 6A,B, the HPLC profiles of the CCSK alkaloids before and after enrichment with XR918C resin both showed three main peaks (a–c). Peaks 1, 2 and 3 were identified as magnoflorine, lindoldhamine and N,N-methyldomesticinium, respectively, by comparing them with the standard samples (Figure 6C–E). It was found that the impurities (with a retention time of about 5.5 min and 16.0 min) were essentially removed after enrichment, indicating a significant improvement in alkaloid concentration. The contents of the primary CCSK alkaloids, including magnoflorine, lindoldhamine and N,N-methyldomesticinium, were then determined (Table 5). The results showed that the enriched product had an alkaloid content of 56.68 ± 0.01% (sum of the three alkaloids), which was 4.62 times higher than that of the crude extract. Additionally, the recovery rate of CCSK alkaloids after XR918C resin enrichment was found to be 92.56 ± 0.01%. In a similar study, Yang et al. [47] used HPD750 resin enrichment for three terpenoid indole alkaloids (vindoline, catharanthine and vinblastine) from *Catharanthus roseus*. The results showed that the enriched product had an alkaloid content of 22.35%, which was 4.03 times higher than that of the crude extract, with a recovery rate of 72.30%. By contrast, XR918C resin exhibited a better enrichment effect for CCSK alkaloids.

### 3.7. Scale-Up Experiment

The scale-up experiment is important for verifying that the process parameters can be used in industrial production. In this study, a 10-fold scale-up in the processing volume of the dynamic enrichment test was performed. Based on the above-obtained optimal process parameters, the CCSK aqueous solution containing 2.45 mg/mL was passed through the XR918C resin column at a flow rate of 2 BV/h. Then, the sample-loaded resin column was washed with 4 BV of water and then eluted with 6 BV of 80% ethanol at a flow rate of 2 BV/h. As mentioned above, the fraction eluted with 80% ethanol was processed to produce a CCSK alkaloids concentrate, which had a content of 54.09 ± 0.03%, 4.41 times higher than that of the crude extract, and a recovery of CCSK alkaloids of 89.19 ± 0.01%. The relative standard deviation (RSD) is a measure of data and process stability. When the RSD is less than 2%, it shows that the data and process are reliable [48]. In this study, an RSD of 0.71% indicated that the results obtained in the scale-up experiment and the optimized process parameters were reliable. Although the purity and recovery rate of CCSK alkaloids enriched after amplification were slightly lower than those of the small batch enrichment, the results meet expectations. The findings indicated that the optimization of process could be used for industrial magnitude enrichment of CCSK alkaloids. The purity and recovery rate of CCSK in the enriched products were both satisfactory.

### 3.8. Regeneration and Recycle

**Figure 7:**
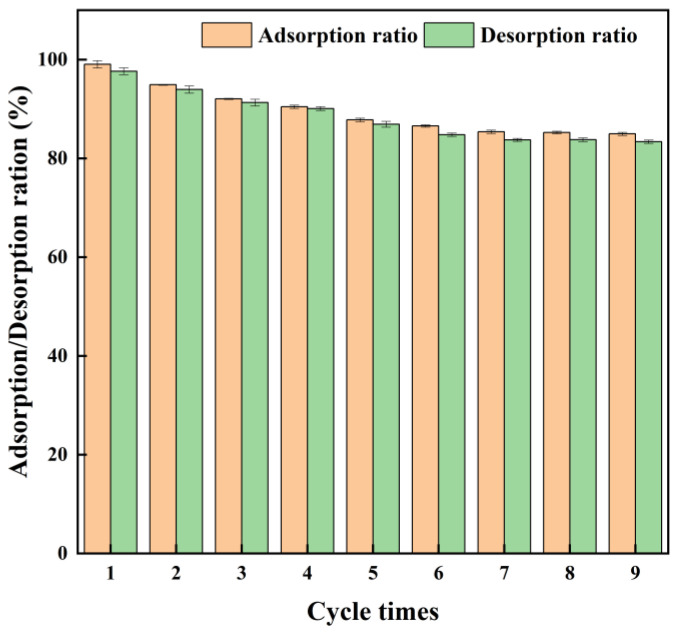
Adsorption and desorption properties of XR918C resin after nine cycles of adsorption and desorption.

The ability of a MAR to be reused and regenerated is the key factor in evaluating its practical application [49]. The sustainability of recovering CCSK alkaloids from XR918C resin was evaluated through continuous fixed-bed adsorption and desorption experiments. The resin was washed with distilled water until the alcohol concentration measured by an alcoholometer was zero, allowing the next adsorption–desorption cycle to be repeated. As shown in Figure 7, the adsorption and desorption rates of XR918C resin remained above 80% after nine cycles of adsorption and desorption. The results demonstrated the high sustainability and potential of XR918C resin for the industrial recycling of alkaloids from the CCSK aqueous solution.

### 3.9. Antioxidant Activities of CCSK Alkaloids Before and After Enrichment

**Figure 8:**
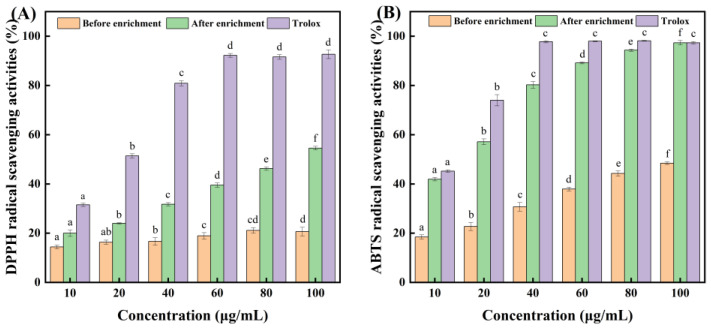
Scavenging activities of DPPH free radicals (**A**) and ABTS free radicals (**B**) of CCSK alkaloids before and after enrichment of XR918C resin. The different letters (a–f) indicate significant differences (*p* < 0.05).

Alkaloids are effective antioxidants that can help prevent the occurrence of some chronic diseases [50]. DPPH is a stable free radical that can act as either a target or a probe for the detection of in vitro antioxidant activity [51]. ABTS is a type of free radical that can be used to measure the total antioxidant capacity of both hydrophilic and lipophilic substances, and it is also frequently used to assess antioxidant activity [52]. Račková et al. [53] found that alkaloids bearing OH moieties showed much better antioxidant activities. In our study, magnoflorine, lindoldhamine and N,N-methyldomesticinium all contained OH moieties, and so these three alkaloids may have good antioxidant activities. Table S2 shows the concentrations of magnolflorine, lindoldhamine and N,N-methyldomesticinium in CCSK alkaloid-enriched samples at different concentrations (10–100 μg/mL). As shown in Figure 8A,B, within the tested concentrations (10–100 μg/mL), the enriched CCSK alkaloids exhibited significantly higher free radical scavenging activities than the unenriched CCSK alkaloids but lower activities than Trolox (the positive control). A dose–response relationship was observed between the sample concentrations and free radical scavenging activities of DPPH and ABTS. At concentrations of enriched CCSK alkaloids between 10 and 100 μg/mL, the ability to scavenge DPPH and ABTS free radicals increased from 20.01% to 54.56% and from 41.93% to 97.34%, respectively. Additionally, the semi-maximum inhibitory concentrations (IC_50_) were calculated by a two-point linear equation connecting the two sides of 50%. The IC_50_ of the enriched CCSK alkaloids for scavenging DPPH and ABTS free radicals were 89.06 μg/mL and 15.30 μg/mL respectively. These values were significantly lower than those of the unenriched samples. Furthermore, there were strong positive correlations between the total CCSK alkaloid contents and their antioxidant activities (*p* < 0.01) (Table 6). These results demonstrated that CCSK alkaloids enriched with XR918C resin exhibited significantly enhanced antioxidant activity.

## 4. Conclusions

This was the first study to enrich alkaloids from CCSKs by MAR technology. XR918C resin was determined to be the most appropriate for the enrichment of CCSK alkaloids (including magnoflorine, lindoldhamine and N,N-methyldomesticinium) by comparison of the adsorption/desorption properties of 16 different types of commercial MARs. Static adsorption equilibrium data were found to be well-described by the pseudo-second-order model and Langmuir isotherm model. The results showed that the adsorption of CCSK alkaloids onto XR918C resin was a spontaneous and exothermic physical reaction. In addition, the establishment of breakthrough and desorption curves were undertaken for the purpose of optimizing the enrichment process. Then, the amplification enrichment experiment (10-fold scale-up in the processing volume of the enrichment test) of alkaloids from CCSK was conducted, and the results showed that the content of total alkaloids increased from 12.26 ± 0.01% to 54.09 ± 0.03%, with a 4.41-fold increase and a recovery rate of 89.19 ± 0.01%. CCSK alkaloids also demonstrated strong DPPH and ABTS free radical scavenging properties after XR918C resin enrichment. In conclusion, the technique developed in this study for enriching alkaloids from CCSK with XR918C resin exhibited significant industrial application potential. However, this study lacks industrial scale-up validation and antioxidant assessment of individual alkaloids, both of which need to be further explored.

## Figures and Tables

**Table 1:** Adsorption kinetics equations and parameters of CCSK alkaloids on XR918C resin.

Models	Equations	Parameters		R^2^
Pseudo-first order	ln(Q_e_ − Q_t_) = −0.1836t + 3.9916	Q_e_ = 54.1414 mg/g	k_1_ = 0.1836 h^−1^	0.9086
Pseudo-second order	t/Q_t_ = 0.00843t + 0.00808	Q_e_ = 118.6240 mg/g	k_2_ = 0.0088 g/(mg·h)	0.9976
Intra-particle diffusion	Q_t_ = 19.2546t^1/2^ + 52.3164	C = 52.3164 mg/g	k_3_ = 19.2546 mg/(g·min^1/2^)	0.9246
(Stage one)	Q_t_ = 39.1345t^1/2^ + 25.3477	C_1_ = 52.8705 mg/g	k_31_ = 39.1345 mg/(g·min^1/2^)	0.9945
(Stage two)	Q_t_ = 19.9081t^1/2^ + 52.8705	C_2_ = 52.8705 mg/g	k_32_ = 19.9081 mg/(g·min^1/2^)	0.9918
(Stage three)	Q_t_ = 19.0845t^1/2^ + 55.2680	C_3_ = 55.2680 mg/g	k_33_ = 19.0845 mg/(g·min^1/2^)	0.8177

**Table 2:** Adsorption isotherm equations and parameters of CCSK alkaloids on XR918C resin.

Models	T (°C)	Equations	Parameters		
			K_L_ (L/mg)	Q_m_ (mg/g)	R^2^
Langmuir	25	C_e_/Q_e_ = 0.00821C_e_ + 3.4469	0.0024	121.80	0.9994
	35	C_e_/Q_e_ = 0.00838C_e_ + 4.2728	0.0020	119.33	0.9968
	45	C_e_/Q_e_ = 0.00853C_e_ + 4.9535	0.0017	117.23	0.9938
			K_F_ [(mg/g)(L/mg)^1/*n*^]	1/*n*	R^2^
Freundlich	25	lnQ_e_ = 0.4107lnC_e_ + 1.6056	4.9809	0.4107	0.9907
	35	lnQ_e_ = 0.4510lnC_e_ + 1.2451	3.4732	0.4510	0.9562
	45	lnQ_e_ = 0.4836lnC_e_ + 0.9663	2.6283	0.4836	0.9664
			A_T_ (L/mg)	K_T_	R^2^
Temkin	25	Q_e_ = 27.6151lnC_e_ − 106.1512	0.0214	27.6151	0.9963
	35	Q_e_ = 28.2561lnC_e_ − 117.0267	0.0159	28.2561	0.9833
	45	Q_e_ = 28.5798lnC_e_ − 123.2985	0.0134	28.5798	0.9891

**Table 3:** Adsorption thermodynamic parameters of CCSK alkaloids on XR918C resin.

T (°C)	lnK_c_	∆G (kJ/mol)	∆H (kJ/mol)	∆S (kJ/mol)
25	6.31	−15.63	−21.73	−20.48
35	6.01	−15.42		
45	5.76	−15.21		

**Table 4:** The fitting parameters of the Thomas, Adams–Bohart and Yoon–Nelson models of CCSK alkaloid adsorption onto XR918C packed column.

Q (BV/h)	Thomas Model			Adams–Bohart Model			Yoon–Nelson Model			
	K_TH_ (mL/mg/min)	Q_e_ (mg/g)	R^2^	K_AB_ (mL/mg/min)	N_0_ (mg/mL)	R^2^	K_Y_ (min^−1^)	τ_cal_ (min^−1^)	τ_exp_ (min^−1^)	R^2^
2.0	0.0059	50.3952	0.9763	0.0030	88.2768	0.8673	0.0130	418.36	410.55	0.9763
3.0	0.0101	43.0866	0.9693	0.0057	72.1501	0.8754	0.0223	238.46	220.17	0.9693
4.0	0.0154	36.3627	0.9429	0.0062	72.9410	0.8912	0.0338	152.22	140.60	0.9429

**Table 5:** Comparison of CCSK alkaloids before and after XR918C resin enrichment.

Type	Magnoflorine (μg/mg)	Lindoldhamine (μg/mg)	N,N-Methyldomesticnium (μg/mg)	Total (μg/mg)	Proportion (%)	Recovery (%)	RSD (%)
Before enrichment	62.46 ± 4.84 ^c^	30.08 ± 4.75 ^b^	30.02 ± 0.20 ^c^	122.56 ± 0.11 ^c^	12.26 ± 0.01 ^c^	-	-
After enrichment	300.31 ± 1.19 ^a^	113.06 ± 5.87 ^a^	153.47 ± 0.53 ^b^	566.83 ± 7.59 ^a^	56.68 ± 0.01 ^a^	92.56 ± 0.01 ^a^	1.33
Scale-up enrichment	269.98 ± 1.57 ^b^	104.37 ± 1.19 ^a^	166.58 ± 1.08 ^a^	540.93 ± 3.83 ^b^	54.09 ± 0.03 ^b^	89.19 ± 0.01 ^b^	0.71

The different letters (a–c) in the same column indicate significant differences (*p* < 0.05). RSD: relative standard deviation.

**Table 6:** Correlation coefficients between the parameters ^a^.

	DPPH	ABTS
CCSK alkaloids before enrichment	0.957 **	0.994 **
CCSK alkaloids after enrichment	1.000 **	0.931 **

^a^ Data represents Pearson correlation coefficient R. ** indicates *p* < 0.01.

## Data Availability

The original contributions presented in this study are included in the article/Supplementary Materials. Further inquiries can be directed to the corresponding authors.

## Supplementary Materials

The following supporting information can be downloaded at: https://www.mdpi.com/article/10.3390/foods15061054/s1, Table S1: The physical properties of different MARs; Table S2: The contents of magnoflorine, lindoldhamine and N,N-methyldomesticinium in CCSK alkaloid-enriched samples at different concentrations (10–100 μg/mL); Figure S1: Correlation spectra of magnoflorine; Figure S2: Correlation spectra of lindoldhamine; Figure S3: Correlation spectra of N,N-methyldomesticinium.

## Abbreviations

The following abbreviations are used in this paper:

AE	Aqueous extraction
CCSK	*Cinnamomum camphora* seed kernel
MAR	Macroporous resin
