# Supplementary material for: Enrichment of Alkaloids from Cinnamomum camphora Seed Kernels Using Macroporous Resin: Adsorption/Desorption Behavior, Process Optimization and Scale-Up Study
Source: Foods. 2026 Mar 17;15(6):1054. doi: 10.3390/foods15061054 (PMC13025361; doi:10.3390/foods15061054)
Supplement: Supplementary file 1 [file foods-15-01054-s001.zip › foods-4148206-supplementary.pdf]

Supplementary Information

Table S1. Physical properties of different MARs.

Type	Particle diameter (mm)	Average pore diameter (nm)	Surface area (m^2/g)	Polarity
YKDH-2	03-1.25	20-22	500-560	Non-polar
X-5	03-1.25	29-31	500-600	Non-polar
HPD-700	03-1.25	8.5-9.0	650-700	Non-polar
HPD-200	03-1.25	8.5-9.0	700-750	Non-polar
LS-300	03-1.25	11-12	700-750	Non-polar
HPD-300	03-1.25	5-5.5	800-870	Non-polar
XR918C	03-1.25	6-9	850-900	Non-polar
HPD-722	03-1.25	13-14	485-530	Weak polar
HPD-BJQH	03-1.25	9-11	600-800	Weak polar
LS-300B	03-1.25	11-12	700-750	Weak polar
XR930C	03-1.25	9-15	700-800	Weak polar
HPD-400	03-1.25	7.5-8.0	500-550	Semi-polar
HPD-450	03-1.25	9-11	500-550	Semi-polar
HPD-750	03-1.25	8.5-9.0	650-700	Semi-polar
NKA-9	03-1.25	15.5-16.5	250-290	Strong polar
YKDH-9	03-1.25	16-18	270-350	Strong polar

Table S2. The contents of magnoflorine, lindoldhamine and N,N-methyldomesticinium in CCSK alkaloid-enriched samples at different concentrations (10-100 μg/mL).

CCSK alkaloid concentrations (μg/mL)	Magnoflorine (μg/mL)	Lindoldhamine (μg/mL)	N,N-methyldomesticinium (μg/mL)
10	2.699	1.044	1.666
20	5.338	2.088	3.332
40	10.796	4.176	6.664
60	16.194	6.264	9.996
80	21.352	8.352	13.328
100	26.990	10.440	16.660

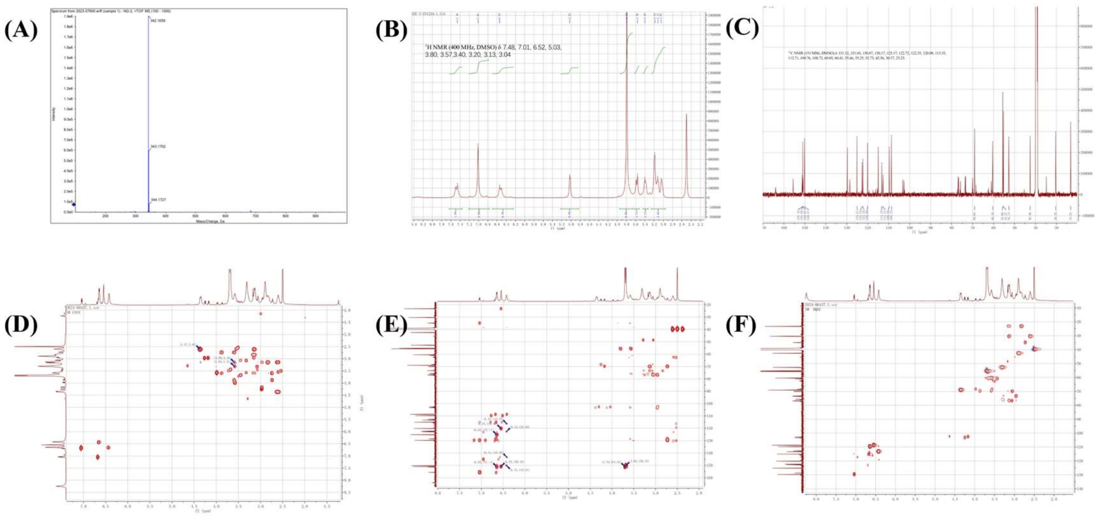

Figure S1. Correlation spectra of magnoflorine. (A) Mass spectrometry. (B) ^{1}H NMR spectrum. (C) ^{13}C NMR spectrum. (D) HSQC spectrum. (E) HMBC spectrum. (F) COSY spectrum.

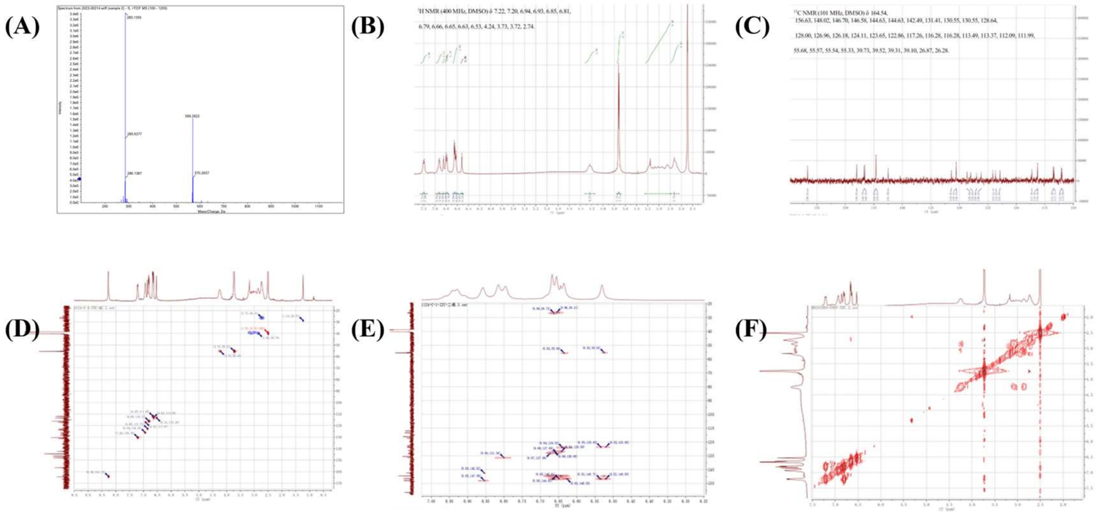

Figure S2. Correlation spectra of lindoldhamine. (A) Mass spectrometry. (B) ^{1}H NMR spectrum. (C) ^{13}C NMR spectrum. (D) HSQC spectrum. (E) HMBC spectrum. (F) COSY spectrum.

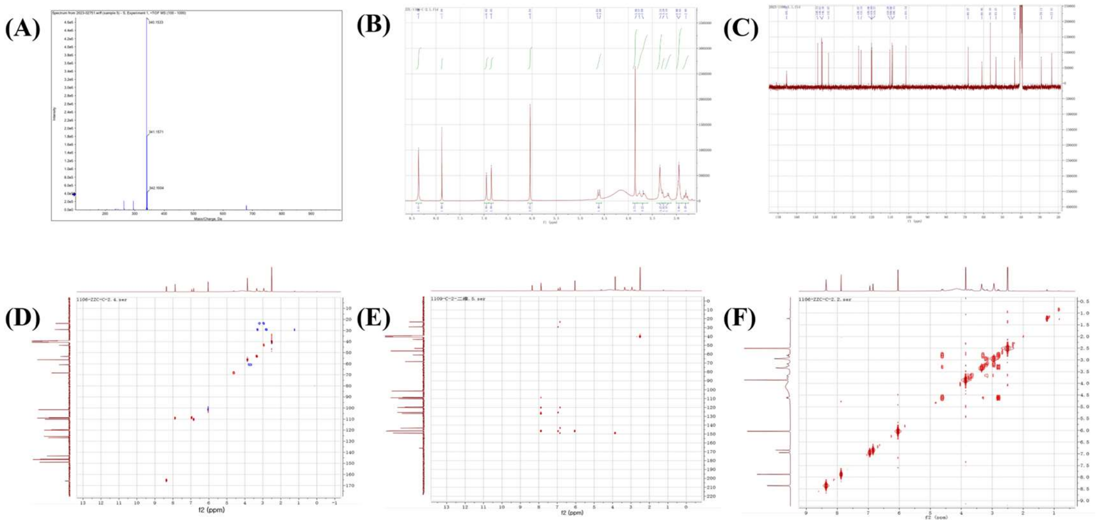

Figure S3. Correlation spectra of N,N-methyldomesticinium. (A) Mass spectrometry. (B) ^{1}H NMR spectrum. (C) ^{13}C NMR spectrum. (D) HSQC spectrum. (E) HMBC spectrum. (F) COSY spectrum.

1.1. The calculation of adsorption/desorption capacity, adsorption/desorption ratio

The adsorption/desorption capacity, adsorption/desorption ratio were calculated by Eq.(S1-S4).

$$Q_e = \frac{(C_0\text{-}C_e)}{W} \times V_i \qquad \text{(S1)}$$

$$A = \frac{(C_0\text{-}C_e)}{C_0} \times 100\% \qquad \text{(S2)}$$

$$Q_d = \frac{C_d V_d}{W} \qquad \text{(S3)}$$

$$D = \frac{C_d V_d}{(C_0\text{-}C_e)V_i} \times 100\% \qquad \text{(S4)}$$

Where Q_e and Q_d (mg/g) represent the adsorption capacity and desorption capacity, respectively; A and D represent the adsorption ratio and desorption ratio, respectively; C_0 (mg/mL) represents the initial alkaloid concentration and C_e (mg/mL) represents the alkaloid concentration at the end of the adsorption process; C_d (mg/mL) represents the alkaloid concentration in the desorption solution; V_i and V_d (mL) represent the volume of the CCSK aqueous solution and the eluate, respectively. W (g) represents the dry weight of the MAR.

1.2. The calculation formula of adsorption kinetics models

The adsorption kinetics of the CCSK alkaloids onto XR918C resin were evaluated using the pseudo-first-order model (Eq. S5), pseudo-second-order model (Eq. S6) and inter-particle diffusion models (Eq. S7):

$$\ln(Q_e - Q_t) = \ln Q_e - K_1 t \quad (S5)$$

$$\frac{t}{Q_t} = \frac{t}{Q_e} + \frac{1}{K_2 Q_e^2} \quad (S6)$$

$$Q_t = K_i t^{1/2} + C_i \quad (S7)$$

Where Q_e (mg/g) represents the total amount of CCSK alkaloids adsorbed at equilibrium, and Q_t represents the total amount of CCSK alkaloids adsorbed onto the resin at t minutes; k_1 (h^{-1}); k_2 (g/(mg min)) denotes the rate constants for the pseudo-first order model and pseudo-second order model, respectively; k_i (mg/(g $min^{1/2}$)) represents the inter-particle diffusion rate constant; C_i (mg/g) is a constant characterized by the thickness of the boundary layer.

1.3. The calculation formula of adsorption isotherms models

The functional relationships between adsorption capacity and alkaloid concentration at different temperatures were analyzed using the Langmuir model (Eq. 8), the Freundlich model (Eq. S9) and the Temkin model (Eq. S10):

$$\frac{C_e}{Q_e} = \frac{C_e}{Q_m} + \frac{1}{Q_m K_L} \qquad (S8)$$

$$\ln Q_e = \ln K_F + \frac{1}{n} \ln C_e \qquad (S9)$$

$$Q_e = K_T \ln A_T + K_T \ln C_e \qquad (S10)$$

Where Q_m (mg/g) refers to the theoretical maximum adsorption capacity; K_L (L/mg) and K_F [(mg/g)(L/mg)$^{1/n}$] refer to the equilibrium constants of the Langmuir model and Freundlich model, respectively; 1/n refers to the adsorption strength of the adsorbent; A_T (L/mg) is the constant coefficient for the Temkin model and K_T (J/mol) refers to the Temkin constant related to the adsorption heat of MAR.

1.4. The calculation formulas of adsorption thermodynamic parameters

The parameters were calculated by Eq. (S11) and Eq. (S12).

$$\triangle G = -RT \ln K_c \qquad (S11)$$

$$\ln K_c = -\frac{\triangle H}{RT} + \frac{\triangle S}{R} \qquad (S12)$$

Where R (8.314 J/mol K), T and K_c represent the gas constant, absolute temperature (K) and equilibrium constant, respectively.
